# Biomechanical optimization of rolling manipulation for lumbar muscle strain: a study protocol of cerebral hemodynamic responses and clinical efficacy using fNIRS

**DOI:** 10.3389/fneur.2026.1850645

**Published:** 2026-09-10

**Authors:** Yuan Yuan, Bo Chen, Xiaojie Su, Qingguang Zhu, Mengni Shi, Yiming Shan, Jintian Chen, Guohui Zhang, Wuquan Sun

**Affiliations:** 1 Department of Tuina, Yueyang Hospital of Integrated Traditional Chinese and Western Medicine, Shanghai University of Traditional Chinese Medicine, Shanghai, China; 2 Department of Traditional Chinese Medicine, Baoshan Branch, RenJi Hospital, School of Medicine, Shanghai Jiao Tong University, Shanghai, China; 3 Research Institute of Tuina, Shanghai Academy of Traditional Chinese Medicine, Shanghai, China; 4 Department of Rehabilitation, Yueyang Hospital of Integrated Traditional Chinese and Western Medicine, Shanghai University of Traditional Chinese Medicine, Shanghai, China

**Keywords:** cerebral hemodynamics, functional near-infrared spectroscopy, lumbar muscle strain, pain-related cortical responses, Tuina rolling manipulation

## Abstract

**Background:**

Lumbar muscle strain (LMS) is a common chronic soft tissue injury disorder, and Tuina, especially Tuina rolling manipulation, is widely used for its management. However, standardized force parameters based on biomechanical effects have not yet been established, and the analgesic mechanisms of rolling manipulation remain insufficiently understood, particularly regarding its influence on cortical hemodynamic responses during manual intervention. Existing studies are fragmented, lacking systematic investigation of the entire chain from pain stimulation-induced cortical hemodynamic responses, to biomechanically optimized manipulation parameters, and to clinical efficacy. Therefore, this study aims to combine functional near-infrared spectroscopy (fNIRS) with biomechanical detection to explore cortical hemodynamic changes induced by pain stimulation, determine the optimal pressure parameters of rolling manipulation and explore the cortical hemodynamic responses associated with rolling manipulation treatment for lumbar muscle strain.

**Methods and design:**

This is a three-part, hierarchical mixed-methods study. Part 1 (Observational): We will compare cerebral hemodynamic responses (via fNIRS) between LMS patients (*n* = 15) and healthy controls (*n* = 15) under two levels of controlled pain stimulation (pain-free vs. high pain threshold). Part 2 (Interventional, Crossover): In 24 LMS patients, we will screen for the optimal pressure level (low, medium, high) of rolling manipulation by evaluating multi-dimensional biomechanical outcomes. Part 3 (Interventional, Controlled): Using the optimal pressure identified in Part 2, we will enroll 69 participants in total, including 46 patients with LMS (23 in the case intervention group and 23 in the case control group) and 23 healthy controls.fNIRS will be used to characterize cortical hemodynamic changes associated with rolling manipulation, while clinical and biomechanical outcomes will be assessed to evaluate therapeutic effects after two treatment courses.

**Discussion:**

This protocol combines neuroimaging and biomechanical assessments to explore the potential association between peripheral biomechanical responses and cortical hemodynamic changes during Tuina rolling manipulation, optimize key operational parameters, and provide preliminary evidence for future clinical research and application.

## 1 Introduction

Chronic lumbar muscle strain, also known as functional low back pain, is a high-incidence musculoskeletal disease, mainly characterized by recurrent soreness and distending pain in the lumbodorsal region (1). It has become a major cause of activity limitation in the working population, and its incidence is increasing year by year and tending to be younger with the changes in modern lifestyles (2). Clinically, commonly used treatment methods included medication and physical therapy (3, 4), among which long-term medication has obvious side effects (5). Tuina therapy in traditional Chinese medicine (TCM) also belonged to physical therapy and has significant advantages. As a classic manipulation, Tuina rolling manipulation could relieve pain and improve local circulation (6, 7). However, current studies are mostly limited to symptom improvement, and the operation relies on the clinician’s clinical experience. Parameters such as pressure and frequency lacked objective quantitative standards, and there is an overall lack of research on cortical responses associated with manipulation and objective standardized mechanical parameters.

Biomechanics provided a quantitative research framework for the standardization of tuina manipulations, which analyzed the characteristics of manipulations from multiple dimensions including kinematics, dynamics and biomechanical effects (8). The stimulation dose of tuina is determined by pressure, frequency and duration, it is not the case that the greater the pressure, the better the effect (9). Excessive or insufficient pressure would affect the curative effect and even cause risks, so it is urgent to standardize the manipulation parameters to ensure safety and effectiveness (10). Kinematic studies have quantified a variety of manipulation parameters, for example, rolling manipulation has four-stage characteristics in operation, among which the operation area is large and the pressure is strong in the 1/5–1/4 time period (11). Dynamic studies have confirmed that the optimal parameter combination of rolling manipulation is a pressure of 4 kg, a frequency of 120 times/min and a duration of 10 min (12), but the quantitative research on manipulations from the perspective of biomechanical effects is still scarce. The research group has previously identified via electromyographic signals that the optimal duration of rolling manipulation for treating lumbar muscle strain was 10 min, which could relieve pain, improve dysfunction and reduce muscle tension (13).

Brain functional imaging techniques reflected the brain functional state by observing changes in brain activity, neural metabolism and hemodynamics, mainly including functional magnetic resonance imaging (fMRI) (14), positron emission tomography (PET) (15), electroencephalogram (EEG) (16), functional near-infrared spectroscopy (fNIRS) (17) and so on. Among them, fMRI has high resolution and could visualize deep brain structures but could not realize real-time detection and is expensive (18). EEG has high temporal resolution, is portable and easy to detect but has poor spatial resolution (19). As an emerging optical technology, fNIRS detects brain changes by detecting the concentration changes of oxyhemoglobin (HbO) and deoxyhemoglobin (HbR), which has the advantages of both temporal and spatial resolution, robustness against motion artifacts, portability, low cost and real-time observation, making it more suitable for the research of tuina manipulations (20, 21). Pain consisted of emotion and sensation and is closely related to the plasticity of central neurons in the brain (22, 23). Therefore, assessing pain-related cortical hemodynamic changes may provide new insights into the central responses associated with low back pain and manual interventions.fNIRS could detect the hemodynamic changes of increased HbO concentration and decreased HbR concentration under neurovascular coupling response (24), and finds that low back pain led to abnormal activation of multiple brain regions such as the dorsolateral prefrontal cortex and reduced related functional connectivity (25, 26), providing a new perspective for understanding the neural mechanism of low back pain.

At present, fNIRS is less applied in the research of tuina manipulations, and its application in the related field of TCM treatment is also scarce. Existing studies have shown that tuina at Hegu acupoint could increase the activation of the primary motor and somatosensory cortex in stroke patients, showing a certain correlation between TCM acupoints and neural circuits (27). Other studies have shown that plantar tuina could induce an increase in prefrontal HbO, suggesting that manual therapy is helpful to enhance sustained attention, verbal short-term and long-term memory (28). However, there is no report on the analysis of fNIRS brain function characteristics of tuina manipulations in the treatment of musculoskeletal diseases, and this research gap limited the in-depth understanding of the neural mechanism of tuina treatment.

### 1.1 Novelty of this study

- This study integrates pain-related cortical responses, biomechanical parameter optimization, and clinical evaluation to provide a systematic framework for investigating the relationship between mechanical stimulation, cortical responses, and therapeutic effects of Tuina rolling manipulation for lumbar muscle strain.
- Based on biomechanical effects, the optimal pressure parameters of rolling manipulation are screened to establish a quantitative correlation system of “individual pain threshold - manipulation mechanical parameters - biological effect”.
- fNIRS technology is first applied to monitor cortical hemodynamic responses during rolling manipulation for lumbar muscle strain, filling the technical gap in investigating cortical hemodynamic responses during TCM tuina interventions.

This study innovatively integrated multiple technologies and indicators: surface electromyography and isokinetic muscle strength are used to screen the optimal pressure parameters of rolling manipulation from the perspective of biomechanical effects and establish quantitative standards. fNIRS technology is applied to tuina research to dynamically monitor changes in cerebral blood oxygen before, during and after intervention, exploring the cortical hemodynamic responses associated with rolling manipulation in the treatment of lumbar muscle strain. fNIRS cerebral blood oxygen indicators, biomechanical indicators and clinical indicators are integrated to construct a multi-dimensional evaluation system for a comprehensive assessment of curative effect, providing a scientific basis for the standardization of rolling manipulation and the precise development of TCM tuina.

## 2 Methods and analysis

### 2.1 Study design and setting

**Figure 1:**
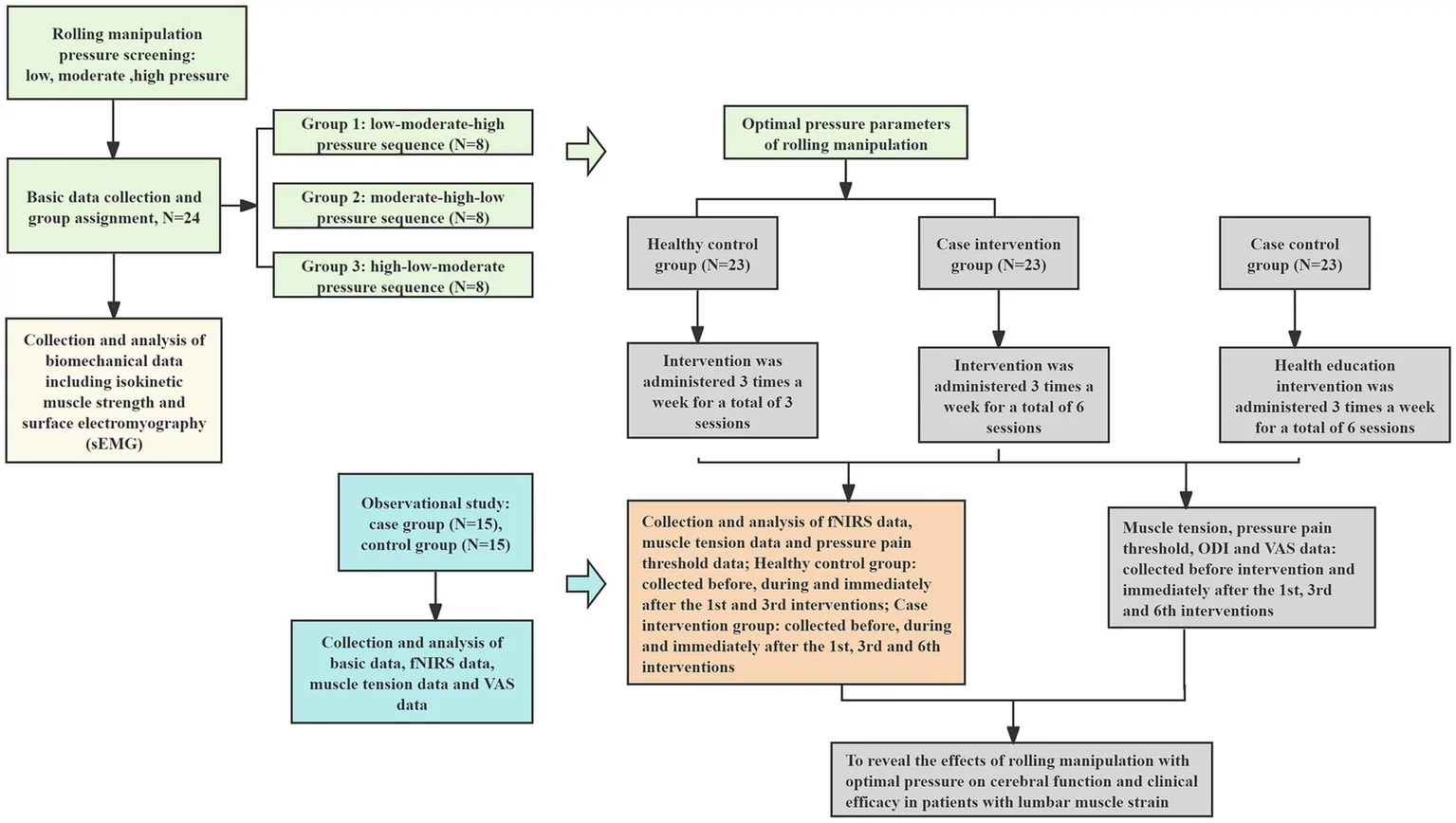
Participant flow chart.

This study consists of three sequential parts, including one observational component and two interventional components. The first part is an observational study aimed at comparing fNIRS-derived cortical hemodynamic responses between healthy controls and patients with lumbar muscle strain under different pain stimulation conditions. The second and third parts are interventional studies aimed at screening the optimal pressure parameters of rolling manipulation and investigating its clinical effects and associated cortical hemodynamic responses. The research setting is mainly based on the clinical testing site with functional near-infrared spectroscopy monitoring conditions and biomechanical detection equipment in the Rehabilitation Medicine Center of Yueyang Integrated Traditional Chinese and Western Medicine Hospital Affiliated to Shanghai University of Traditional Chinese Medicine. Equipment such as rapid muscle condition determinator, simulated dynamometer, surface electromyography, and isokinetic muscle strength testing system are used for data collection. Intervention methods included pain stimulation of different intensities (pain-free range and high pain threshold) and TCM rolling manipulation with graded forces (low, medium and high). This study protocol followed the SPIRIT 2013 Statement for Reporting Clinical Trial Protocols and the ethical principles proposed in the Declaration of Helsinki (2013 version). It has been approved by the Ethics Committee of Yueyang Integrated Traditional Chinese and Western Medicine Hospital Affiliated to Shanghai University of Traditional Chinese Medicine (Approval No.: 2025–197-01), all sub-centers have completed the ethical review process, and it has been registered on the International Clinical Trial Registry Platform for Traditional Medicine (Registration No.: ITMCTR2025001823). The design process is shown in Figure 1.

### 2.2 Participants

#### 2.2.1 Inclusion criteria

The research subjects of this study are divided into a case group and a control group. The case group consists of patients who met the diagnostic criteria for lumbar muscle strain in the General Principles for the Formulation and Revision of Diagnostic and Efficacy Evaluation Criteria for TCM Diseases and Syndromes (No.: ZY/T 10–2024) issued by the National Administration of Traditional Chinese Medicine in 2024 (29). The case groups in the first and third parts needed to meet the following criteria: aged 18–69 years old, regardless of gender; recurrent low back pain for more than 3 months; no other treatment for low back pain received 3 weeks before enrollment; the core area of muscle pain confirmed by physical examination is located in the lumbar erector spinae, ranging from the paravertebral region of the 12th thoracic spinous process to the dorsal surface of the sacrum and the posterior part of the iliac crest; voluntary signing of the informed consent form and cooperation in completing the entire study and providing relevant data. The second part focused on the screening of tuina manipulation pressure, so confounding factors needed to be strictly controlled, and additional requirements are made on the basis of the above criteria: aged 20–40 years old, body mass index (BMI) controlled at 18.5-24 kg/m^2^. The control group is healthy people of the same age group as the case group in the corresponding research part (18–69 years old for the first and third parts, no control group for the second part), without lumbocrural pain symptoms and a history of neuromuscular diseases, who voluntarily signed the informed consent form and cooperated in completing relevant tests.

#### 2.2.2 Exclusion criteria

The contents of the case group and the control group are consistent, and the three parts of the experiment are identical, except that Item (h) is absent in the second part. (a) Patients with mental disorders or impaired consciousness; (b) Pregnant, lactating women and women preparing for pregnancy; (c) Heavy smokers, defined as those smoking at least 20 cigarettes (1 pack) per day; (d) Complicated with non-soft tissue chronic injury diseases such as lumbar intervertebral disc herniation, spinal stenosis and intraspinal lesions; (e) Pain caused by lumbar spine surgery, trauma, rheumatoid arthritis, etc.; (f) Malignant tumors, acute infectious diseases, severe skin diseases, rheumatism and immune diseases, complicated with severe heart, liver and kidney diseases; (g) Patients participating in other external therapies, medications and other treatments that may affect the judgment of results; (h) Contraindications to fNIRS monitoring such as a history of craniocerebral surgery, cranial defects and phobia of headgear wearing.

#### 2.2.3 Criteria for elimination, termination and attrition

The contents of the case group and the healthy group are consistent, and the three parts of the experiment are identical. (a) Those who did not meet the inclusion/exclusion criteria or concealed their medical history after enrollment; (b) Those who failed to follow the prescribed intervention measures and showed poor compliance; (c) Those who developed serious adverse reactions during the intervention, or experienced exacerbation of underlying diseases and certain complications; (d) Those who withdrew voluntarily or lost contact.

### 2.3 Recruitment, randomization and blinding

#### 2.3.1 Recruitment

This study is conducted in the outpatient departments of Tuina and Rehabilitation of Yueyang Integrated Traditional Chinese and Western Medicine Hospital Affiliated to Shanghai University of Traditional Chinese Medicine. Recruitment announcements are released through the hospital’s official social media accounts to maximize the visibility and accessibility of potential participants. Subjects are recruited on a voluntary basis, and all participants have to voluntarily sign the informed consent form and provide relevant information and data.

#### 2.3.2 Randomization and blinding

Randomization is standardized in this study in combination with the design characteristics of the three parts of the experiment: The first part is a case–control observational study. After screening through strict inclusion and exclusion criteria, the case group (patients with lumbar muscle strain) and the control group (healthy people without lumbocrural pain) are selected from the eligible population by simple random sampling (15 cases in each group) to ensure sample representativeness, with no inter-group random allocation involved. The second part is a cross-over design study, in which 24 patients with lumbar muscle strain are divided into 3 groups (8 cases in each group) by complete randomization, receiving three sequences of rolling manipulation intervention with low-medium-high, medium-high-low and high-low-medium pressure, respectively. The third part is an interventional trial involving 69 participants in total. 46 patients with lumbar muscle strain are stratified by age and disease duration and then divided into a case intervention group (rolling manipulation intervention, *n* = 23) and a case control group (health education intervention, *n* = 23) by the random number table method; additionally, 23 healthy controls are selected by simple random sampling.

This study adopts a partial blinding method to control bias, and supplements standardized training for operators to reduce operation bias: At the participant level, in the crossover design of the second part, participants are unaware of the pressure level and intervention sequence. In Part 3, participant blinding to the type of intervention is not feasible because the case intervention group receives manual rolling manipulation whereas the case control group receives health education. Participants are not informed of the study hypotheses or the expected superiority of either intervention. Outcome assessors and statistical analysts remain blinded to group allocation. Data collection is performed by full-time researchers who do not participate in the interventions, and statistical analysts receive coded anonymous data; neither is aware of the grouping information. All operators receive unified standardized training for 3 times (2 h each time), including the calibration of manipulation pressure, frequency and operation position, and only pass the assessment (the consistency of manipulation parameters ICC>0.8) can they perform the intervention independently. Blinding is maintained by separating the intervention and data collection settings and formulating confidentiality protocols. The blind is only broken in accordance with procedures and documented in detail upon approval by the ethics committee in special circumstances such as the occurrence of serious adverse reactions in participants.

### 2.4 Interventional measures

#### 2.4.1 Part 1: cortical hemodynamic responses to pain stimulation

First, a rapid muscle condition determinator is used to measure the muscle tension of the left and right lumbar erector spinae and quadratus lumborum of the patients. Then, pressure pain threshold detection is performed using a simulated dynamometer, and the pain degree is divided into a pain-free range (with a touch feeling, VAS 0–3 points) and a high pain threshold (painful but tolerable, VAS 7–10 points). After the pressure pain threshold measurement, the subjects are pressurized according to the obtained values, and functional near-infrared spectroscopy (fNIRS) is used for detection while pressurization is applied: baseline measurement with 6 min of rest before pain intervention; during pain intervention, 30s of pain-free range - 30s of recovery, for 5 cycles; 30s of high pain threshold - 30s of recovery, for 5 cycles; a 6-min resting measurement is conducted after all interventions, and data are collected.

Muscle tension detection points (selected according to the body surface projection of muscles): bilateral erector spinae: marked points at the muscle belly eminence 3 cm beside the 4th lumbar spinous process.

Bilateral quadratus lumborum: marked points at the muscle belly eminence 3 cm beside the 3rd lumbar spinous process at the level between the posterior iliac crest and the 12th rib.

Bilateral pressure pain threshold detection points: marked points at the muscle belly eminence 3 cm beside the 4th lumbar spinous process.

#### 2.4.2 Part 2: screening optimal rolling manipulation pressure

The low, medium, and high pressure levels were determined using the following procedure. Incremental pressure was applied to the marked points of the bilateral lumbar erector spinae using a simulated dynamometer (NK-500). Participants reported their perceived sensation according to the VAS. Pressure values corresponding to VAS 0–3 (low), 4–6 (medium), and 7–10 (high) were recorded separately for the left and right sides as individual reference values. Before the formal measurement, all participants received standardized training on the VAS scoring criteria, with clear explanations of the sensory descriptors corresponding to 0–3, 4–6, and 7–10. The measurement was started only after the participant confirmed their understanding. Each pressure level was measured three times with a 30-s interval, and the mean value was used for subsequent analysis. All measurements were performed by the same trained operator.

A two-way repeated-measures ANOVA was then performed on these reference values to examine the effects of side, pressure level, and their interaction. If neither the side effect nor the interaction reached statistical significance, the reference values of the corresponding pressure levels were pooled across sides; otherwise, the sides were analyzed separately. A one-way repeated-measures ANOVA was then used to evaluate whether the three predefined pressure levels show statistically distinguishable force values on the pooled data. If no significant difference was found, the classification of pressure levels would be re-evaluated.

The fluctuation ranges of the low, medium, and high pressure levels were calculated separately using the 95% confidence intervals: Fluctuation range (%) = (upper limit of 95%CI − lower limit of 95%CI) / (2 × mean) × 100%. The fluctuation range value (in Newtons) was then calculated as the mean multiplied by the fluctuation range percentage. For each pressure level, the lower limit of the interval was calculated as the mean minus the fluctuation range value, and the upper limit of the interval was calculated as the mean plus the fluctuation range value. The high-pressure level was applied strictly within the participant’s pain tolerance, and the operator’s physical capacity was also considered to avoid injury. After the pressure levels were determined, the Novel Pliance-X 32 Expert glove-type pressure distribution measurement system was used to measure the mechanical parameters of the rolling manipulation as an operational reference and to monitor standardization. The system is calibrated once a day before the operation to ensure the accuracy of pressure measurement. After the tuina pressure range is set, subsequent patients are strictly screened according to the “tuina pressure value range”determined by the experiment, and only subjects whose lumbar erector spinae bearing threshold fell within this range are included, and those beyond the range (too high/too low) are directly excluded.

A preliminary study was conducted before the formal trial to determine the washout duration. Six participants with lumbar muscle strain received a single session of rolling manipulation using clinically common parameters (4 kg, 120 times/min, 10 min). The root mean square (RMS) values of surface electromyography (sEMG) from the bilateral erector spinae were recorded before the intervention, immediately after the intervention, and on Days 3, 5, and 7 after the intervention. The RMS values increased immediately after the intervention, declined by Day 3, fell below the pre-intervention level by Day 5, and remained stable on Day 7. No statistically significant differences were observed between the pre-intervention values and those on Days 5 and 7, indicating that muscle activity had reached a stable state within 7 days. Accordingly, the washout period was set to 7 days (Table 1).

**Table 1:** RMS values of the bilateral erector spinae during the washout observation (*n* = 6).

Side	Baseline	Immediately after intervention	Day 3	Day 5	Day 7
Left (μV)	48.39 ± 19.42	54.37 ± 19.45*	49.64 ± 11.10	42.16 ± 8.67	41.16 ± 4.58
Right (μV)	45.46 ± 14.29	49.92 ± 23.50	48.16 ± 13.46	42.33 ± 11.37	43.10 ± 15.68

*p < 0.05 vs. baseline. RMS: root mean square, reflecting the intensity of muscle activation. RMS increased immediately after the intervention, declined by Day 3, fell below the baseline level by Day 5, and remained stable on Day 7. The statistically significant increase immediately after the intervention was observed on the left side.

Participants were randomly assigned to one of three intervention sequences: low-medium-high, medium-high-low, or high-low-medium. Taking the low-medium-high sequence as an example, the procedures for the other two sequences (medium-high-low and high-low-medium) were identical, with the only difference being the order of pressure application.

First, surface electromyography, isokinetic muscle strength, muscle tension, pressure pain threshold and VAS indicators of the subjects’ bilateral lumbar regions are detected before the test; then, low-pressure rolling manipulation stimulation is applied to the subjects’ lumbar regions, with the intervention position at the bilateral L1-L5 lumbar erector spinae, repeated intervention from bottom to top for 10 min at 120 times/min; the operators are required to have more than 5 years of clinical experience in TCM tuina and hold a TCM practicing physician certificate. Finally, surface electromyography, isokinetic muscle strength, muscle tension, pressure pain threshold and VAS indicators of the bilateral lumbar regions are detected immediately after the rolling manipulation intervention.

Second, after the completion of the low-pressure rolling manipulation stimulation operation procedure and detection, the subjects enter a washout period. After the end of the washout period, the subjects receive medium-pressure rolling manipulation stimulation, and the operation procedure is the same as that of the low-pressure rolling manipulation stimulation, with the same detection methods and processes for medium and low pressure.

Finally, after the completion of the medium-pressure rolling manipulation stimulation operation procedure and detection, the subjects enter a washout period. After the end of the washout period, the subjects receive high-pressure rolling manipulation stimulation, and the operation procedure is the same as that of the low-pressure rolling manipulation stimulation, with the same detection methods and processes for high and low pressure.

The primary outcomes for screening the optimal pressure are the changes in the surface electromyographic (sEMG) indicators of the bilateral lumbar erector spinae, including the time-domain indicator (root mean square, RMS) and the frequency-domain indicator (median frequency, MF). The final determination of the optimal pressure level will be made by combining these primary outcomes with secondary biomechanical indicators, including isokinetic muscle strength, muscle tension, and pressure pain threshold. The outcomes will be analyzed separately. Differences among the low-pressure, medium-pressure, and high-pressure conditions will first be examined for each outcome. For outcomes showing significant differences across pressure levels, regression analysis will be further performed to examine how the outcomes change across different pressure levels (as detailed in Section 3.10). Correlation analyses will also be performed to examine the relationships among the different biomechanical measures. The final pressure will be selected according to the overall pattern of the sEMG and other biomechanical findings within the predefined VAS tolerance range. A favorable change in a single secondary outcome alone will not determine the optimal pressure.

#### 2.4.3 Part 3: clinical efficacy and cortical hemodynamic responses with optimal pressure

The experiment is divided into three groups, with healthy individuals without lumbocrural pain serving as the healthy control group, who receive intervention using optimal pressure parameters of the rolling manipulation. The protocol is 3 times per week, with one week as one treatment course, for a total of one course (3 interventions). This intervention is administered to investigate cortical hemodynamic changes induced by rolling manipulation with optimal pressure and to assess neuromuscular responses in healthy individuals with intact lumbar muscles, enabling a direct comparative analysis of brain functional and biomechanical responses to the identical manual intervention between healthy subjects and patients with lumbar muscle strain. fNIRS data are collected immediately before, during and after the 1st and 3rd interventions, while muscle tension and pressure pain threshold data are obtained before and after the intervention. Patients with lumbar muscle strain are divided into two groups: a case control group and a case intervention group. The case control group receives health education, whereas the case intervention group receives optimal-pressure rolling manipulation. The health education mainly covers posture management in daily life and work, lumbar protection, and guidance on appropriate physical activity and exercise, with participants’ questions addressed as needed. For both groups, the intervention protocol is 3 times per week, with one week as one treatment course, for a total of two courses. Muscle tension, pressure pain threshold, the Oswestry Disability Index (ODI) and VAS scores are assessed before intervention and immediately after the 1st, 3rd and 6th interventions, at the same detection positions as in Part 1. For the case intervention group, fNIRS data are additionally collected before, during and after the 1st and 3rd interventions. During fNIRS acquisition, participants remained in a prone position with the head and upper chest supported by soft pillows, and the forearms crossed under the forehead to provide additional head support. This posture ensured that the head remained in a relaxed and stable position. The rolling manipulation was applied exclusively to the lumbar region using a smooth, continuous rolling technique without abrupt flicking or plucking motions, thereby minimizing mechanical transmission to the head. The enrollment schedule and assessment protocol are shown in Table 2.

**Table 2:** Study schedule.

Stage	First visit	Pre-intervention	Intervention	Intervention	Post-intervention
Sign informed consent form	X				
Assign group number	X				
Lumbar spine AP & lateral X-ray	X				
Collect basic medical history					
General information	X				
Specialized examination	X				
Clinical DIAGNOSIS	X				
Confirm inclusion criteria	X				
Confirm exclusion criteria	X				
Efficacy observation part 1 trial		Pre-intervention	Intervention	Post-intervention	
Functional near-infrared spectroscopy (fNIRS)		X	X	X	
Muscle tension		X		X	
Pressure pain threshold		X		X	
Visual analogue scale (VAS)			X		
Part 2 trial		Pre-intervention	1st Post-intervention	2nd Post-intervention	3rd Post-intervention
Surface electromyography (sEMG)		X	X	X	X
Isokinetic muscle strength		X	X	X	X
Muscle tension		X	X	X	X
Pressure pain threshold		X	X	X	X
Visual analogue scale (VAS)		X	X	X	X
Part 3 trial		Pre-intervention	1st Intervention	3rd Intervention	6th Intervention
Oswestry disability index (ODI)		X	X	X	X
Functional near-infrared spectroscopy (fNIRS)			Before-during-after	Before-during-after	
Muscle tension		X	X	X	X
Pressure pain threshold		X	X	X	X
Visual analogue scale (VAS)		X	X	X	X
Study evaluation					
Treatment compliance	X				X
Adverse event assessment		X			X
Analysis of dropout reasons					X
Study completion summary					X
CRF statement review					X

To standardize the measurement conditions, all participants in Parts 1, 2, and 3 of the study were asked to maintain their usual daily activity level during the study period and to avoid additional strenuous physical activity. In addition, before each measurement, participants rested in a quiet environment for at least one hour to minimize the acute effect of physical activity on the baseline values.

### 2.5 Outcome measures

#### 2.5.1 General information collection

General information included age, gender, height, weight, BMI, waist-hip ratio, heart rate and medical history, smoking status, and habitual physical activity level.

#### 2.5.2 Primary outcome measures

**Figure 2:**
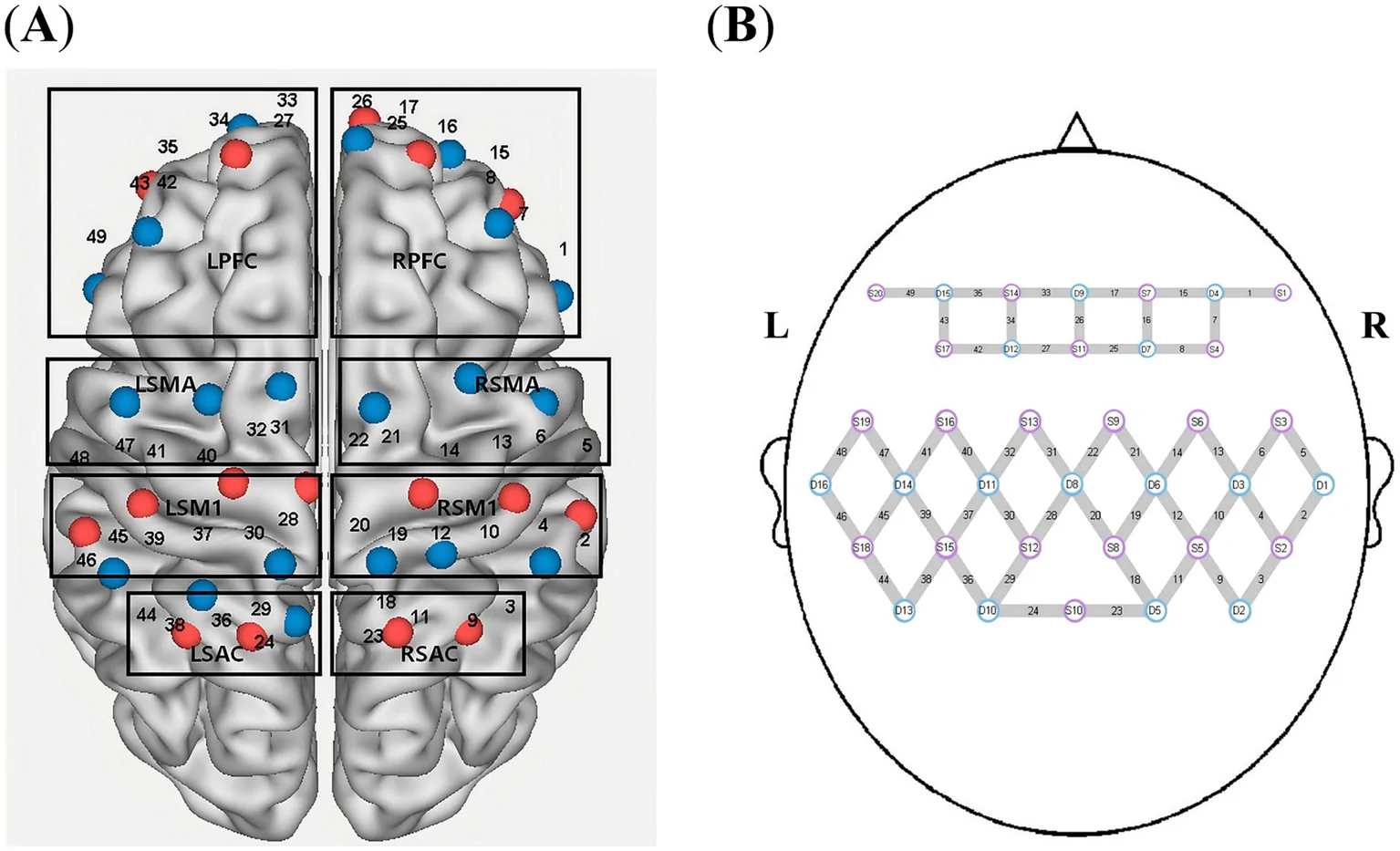
fNIRS probe placement and channel distribution map. Three-dimensional and two-dimensional representations of fNIRS channel distribution. **(A)** Three-dimensional model showing cortical regions and fNIRS channel distribution. **(B)** Two-dimensional channel distribution model. L, left; R, right; LPFC, left prefrontal cortex; RPFC, right prefrontal cortex; LSMA, Left supplementary motor area; RSMA, right supplementary motor area; LSM1, left primary motor cortex; RSM1, right primary motor cortex; LSAC, left somatosensory association cortex; RSAC, right somatosensory association cortex.

Functional near-infrared spectroscopy (fNIRS) detection (Part 1): The primary outcome is the cortical hemodynamic response measured by fNIRS, expressed as changes in oxygenated hemoglobin (HbO) and deoxygenated hemoglobin (HbR). Measurements are obtained during the pain-free and high-pain stimulation conditions and compared with the pre-stimulation baseline. The primary comparison is between patients with LMS and healthy controls under the two stimulation conditions. We hypothesize that the cortical hemodynamic responses to pressure stimulation differ between the two groups. The fNIRS system used is a portable 49-channel system manufactured by HuiChuang (Danyang, China), consisting of 20 light sources and 16 detectors. The system uses two wavelengths of 730 and 850 nm, with a sampling rate of 11 Hz and a fixed source-detector separation of 3 cm. The probe array was positioned according to the international 10–10 system, with the D8 optode of the array aligned to the Cz position. Optode positions are recorded for each participant using a three-dimensional digitizing system and registered to Montreal Neurological Institute (MNI) space. The 49 channels cover eight predefined cortical regions: the left and right prefrontal cortex (LPFC and RPFC), left and right supplementary motor area (LSMA and RSMA), left and right primary motor cortex (LSM1 and RSM1), and left and right somatosensory association cortex (LSAC and RSAC) (Figure 2), and recorded data from participants in the static prone position and under different pain stimulations (pain-free range, VAS = 0–3; high pain threshold range, VAS = 7–10). Data preprocessing was performed using Nirspark V1.8.9 software. The preprocessing procedure included channel quality assessment, motion artifact identification and correction, band-pass filtering, conversion of optical density signals to hemoglobin concentration, and baseline correction. Channels with poor signal quality were identified according to the software-based quality control procedure and excluded from further analysis. Motion artifacts were automatically detected and corrected using the built-in motion correction algorithm, with a standard deviation threshold of 6 and amplitude threshold of 0.5. A band-pass filter (0.01–0.1 Hz) was applied to remove physiological noise and low-frequency drift. The modified Beer–Lambert law was used to calculate HbO and HbR concentrations with a differential pathlength factor (DPF) of 6. For the pain-stimulation task in Part 1, task-related hemodynamic responses are analyzed using the general linear model (GLM) implemented in NirSpark. The stimulus function is modeled using the built-in hemodynamic response function (HRF), which is defined in the software by two gamma functions separated by 2 s. The resulting β coefficients of HbO and HbR are used for subsequent analysis. For resting-state and continuous rolling-manipulation data, HbO time series are analyzed using functional connectivity, wavelet amplitude, and graph-theoretical measures. In Part 3, these analyses are used to characterize cortical hemodynamic changes before, during, and after optimal-pressure rolling manipulation.

Surface electromyographic signal detection (Part 2): The primary outcomes are the changes in sEMG RMS and MF from before to immediately after each rolling manipulation session. The primary comparison is the difference in these changes among the low-pressure, medium-pressure, and high-pressure conditions within the same participants. We hypothesize that the three pressure levels produce different immediate sEMG responses. The Noraxon Ultium surface electromyography system was used. The subjects took a prone position, and the electrode patches were attached to the marked points of the bilateral lumbar erector spinae and quadratus lumborum. The sEMG signals were recorded before and immediately after each rolling manipulation session with different pressure levels. The time-domain indicator (RMS) and the frequency-domain indicator (MF) were selected as the primary electromyographic outcomes for identifying the optimal pressure level. The RMS reflects the intensity of muscle activation, and the MF reflects muscle fatigue status.

Oswestry Disability Index (ODI) (Part 3): The primary clinical outcome is the Oswestry Disability Index (ODI). The main analysis will compare the change in ODI from baseline to immediately after the 6th intervention between the case intervention group and the case control group. We expect a greater reduction in ODI in the case intervention group. ODI will also be assessed after the 1st and 3rd interventions to describe the course of clinical change. It covered 10 dimensions including pain intensity, activities of daily living, walking, sitting and sleeping, with 0–5 points for each dimension. The total score is converted into a percentage (0–100%), with a higher score indicating more severe dysfunction. Data are collected before intervention and immediately after the 1st, 3rd and 6th interventions to evaluate improvements in patients’lumbar function. If no more than 1 item is missing, the value is replaced by the average score of the completed items; if 2 or more items are missing, the data are regarded as invalid.

#### 2.5.3 Secondary outcome measures

Muscle tension detection at marked points of bilateral lumbar erector spinae and quadratus lumborum (Part 1,2,3): A rapid muscle condition measurement system (OE-220, ITO, Japan) is used. The detection positions are the bilateral erector spinae (muscle belly eminence 3 cm beside the 4th lumbar spinous process) and bilateral quadratus lumborum (3 cm beside the 3rd lumbar spinous process at the level between the posterior iliac crest and the 12th rib). The probe is pressed vertically on the marked points until a “beep” is heard, then the reading is taken, repeated 3 times (30s interval) to take the average value, and detected at each time node of pain stimulation intervention, rolling manipulation intervention with different pressure and rolling manipulation intervention with optimal pressure.

Pressure pain threshold detection at marked points of bilateral lumbar regions (Part 1,2,3): A simulated dynamometer (NK-500, ALGOL, Japan) is used, with the detection point at the muscle belly eminence 3 cm beside the 4th lumbar spinous process. The test head is pressurized at a constant rate and supervised by VAS score. When the subject reaches the corresponding pain threshold, he/she reported a pause and the data is recorded, repeated 3 times (30s interval) to take the average value, and detected synchronously with each stage of intervention throughout the process.

Visual Analogue Scale for Pain (VAS) (Part 1,2,3): The pain degree is evaluated by a 10-scale sliding ruler. 0–3 points is the pain-free range/low pressure range, 4–6 points is the moderate intensity pain/medium pressure range, and 7–10 points is the high pain threshold/high pressure range. It is used to supervise the intensity of pain stimulation, define the pressure of rolling manipulation and evaluate the pain relief effect after each stage of intervention, and recorded in real time at each intervention node.

Isokinetic muscle strength testing (Part 2): An isokinetic dynamometer (Biodex System-III, Biodex Medical Systems, Shirley, NY, USA) was used for the test. The participant was seated on the Biodex back testing chair with the trunk upright, the pelvis and thighs secured with straps, and the feet not supported by the footplate to avoid lower limb involvement. The axis of the dynamometer was aligned with the L5 vertebra. The test was performed at angular velocities of 60°/s and 120°/s with five repetitions each, during which the participant performed maximal voluntary lumbar flexion and extension. The measured parameters included peak torque (PT), peak torque to body weight ratio (PT/BW), average power (AP), and flexion/extension ratio (F/E). Isokinetic muscle strength data were collected before and immediately after each intervention session, serving as a secondary biomechanical indicator for the final determination of the optimal pressure level. In addition, sEMG signals from the bilateral erector spinae and quadratus lumborum were recorded simultaneously during the isokinetic test, with electrode placement identical to that described in the surface electromyography section.

### 2.6 Participant safety

The inclusion and exclusion criteria are strictly implemented, contraindications are excluded through medical history collection, physical examination and imaging examination, and the informed consent form is signed with the right of voluntary withdrawal clearly stated. During the intervention, the VAS score is used to define the range of pain stimulation and rolling manipulation pressure, and the participants’ feelings are continuously monitored. If discomfort occurs, the intervention is immediately stopped and handled. Tuina operations are performed by professional doctors who have passed standardized training and hold relevant qualification certificates, in accordance with standardized procedures, and manipulation parameters are calibrated by relying on instruments. All detection equipment is regularly maintained, and qualified consumables are selected. An adverse event recording and emergency response mechanism is established. For better prevention and treatment, researchers detected any potential adverse events and recorded them truthfully on the Case Report Form (CRF).

### 2.7 Quality control

Random grouping and control design are adopted, the basis for sample size calculation is clarified, a standardized research manual is formulated, and all researchers take up their posts only after passing pre-job training. A supervision group and inspectors are set up, subjects are strictly screened during recruitment, and baseline data between groups are calibrated. For the crossover design in Part 2, the 7-day washout period duration is specifically justified by preliminary observation data to minimize potential carryover effects between successive interventions. Intervention operations are performed by fixed personnel, and manipulations and instruments are regularly calibrated. Detection data follow unified operating specifications, a double entry and review system is adopted, and missing and abnormal data are handled in accordance with regulations. Appropriate methods are selected for statistical analysis with multiple comparison correction, which is completed by professionals with analysis codes retained. Paper and electronic data are managed in a standardized manner throughout the process to ensure research traceability and reliable results.

### 2.8 Sample size calculation

Based on the core research objectives and design types of the three parts, this study is overall planned as follows:

The first part is an observational study, with the fNIRS-derived cortical hemodynamic response (HbO/HbR changes) as the primary outcome. Based on repeated measures analysis of variance (effect size *f* = 0.286, α = 0.05, 1−β = 0.8) (30), G*Power 3.1.9.7 calculation showed that 12 cases are needed in each group. Considering a 20% attrition rate, 15 cases are included in both the healthy control group without lumbocrural pain and the lumbar muscle strain case group, with a total of 30 cases.

The second part is a cross-over design. The sample size was estimated based on the median frequency (MF) of the surface electromyographic (sEMG) signals, one of the primary electromyographic outcomes for screening the optimal pressure. Referring to our previous study on rolling manipulation in patients with lumbar muscle strain (13), the within-individual standard deviation of sEMG frequency was 17.09 Hz and the intraclass correlation coefficient was 0.5. Repeated Measures ANOVA combined with Greenhouse–Geisser corrected F test (α = 0.05, 1-β = 0.8) was adopted. PASS 2021 software calculation showed that 17 cases are needed. Considering a 20% attrition rate, 24 patients with lumbar muscle strain are included and randomly divided into 3 group sequences.

The third part took the change in the Oswestry Disability Index (ODI) from baseline to immediately after the 6th intervention as the primary clinical endpoint for the comparison between the case intervention group and the case control group. Based on a two-independent-sample design, in which the expected between-group difference (μ2 − μ1 = 10) and the standard deviation (σ = 9) were derived from a previous study on massage combined with functional exercise for chronic lumbar muscle strain (31), With α = 0.05 and 1 − β = 0.80, the calculation showed that 18 patients were required in each group. Considering a 20% attrition rate, 23 patients with LMS will be enrolled in the case intervention group and 23 in the case control group, resulting in a total of 46 patients with LMS. In addition, 23 healthy controls will be enrolled as a reference group for the fNIRS and biomechanical comparisons. Therefore, Part 3 will include 69 participants in total, comprising 46 patients with LMS and 23 healthy controls.

### 2.9 Data collection and management

Data collection in this study ran through the entire process of the three parts of the research, following the principles of standardization and real-time, and data management focused on integrity, accuracy and security. In terms of data collection, a standardized designed Case Report Form (CRF) is uniformly used to record the subjects, baseline information, intervention-related data and detection results of each outcome indicator. Among them, instrument detection data such as fNIRS, surface electromyography and isokinetic muscle strength are exported in real time and marked with detection time points and subject numbers, and manually recorded data have to be recorded promptly after the completion of detection to ensure no information omission. In the data management link, a dual-track storage system of electronic database and paper files is established. Electronic data are entered with encryption software and regularly backed up off-site, and paper CRF forms are filed in special filing cabinets by number. A double entry and review system is implemented, with cross-checking after entry completion. If contradictory data are found, the original detection records are traced in a timely manner for verification and correction. The rules for handling missing data were clarified, and abnormal data have to be marked with reasons and reviewed by the research person in charge. The privacy protection norms of medical research are strictly followed throughout the process. All data are only used for the analysis of this study, the personal information of subjects is anonymized, and private disclosure or transmission is prohibited to ensure that the data is traceable, verifiable, safe and confidential throughout the process.

### 2.10 Statistical analysis

Statistical analyses will be performed using MATLAB R2024b. fNIRS preprocessing and calculation of HbO/HbR concentrations, functional connectivity (FC), wavelet amplitude (WA), and graph-theoretical measures will be performed using NirSpark V1.8.9. Normally distributed continuous variables will be expressed as mean ± standard deviation, non-normally distributed variables as median and interquartile range, and categorical variables as frequencies and percentages. Normality will be assessed using the Shapiro–Wilk test. *p* < 0.05 will be considered statistically significant.

Baseline data: Baseline variables will be analyzed according to variable type, data distribution, and the number of groups using the t test, analysis of variance (ANOVA), Mann–Whitney U test, Kruskal-Wallis test, or chi-square test, as appropriate.

fNIRS data: Task-related fNIRS data will be analyzed using the general linear model (GLM) implemented in NirSpark to obtain channel-wise HbO and HbR β coefficients for subsequent group comparisons. To reduce the risk of false-positive findings arising from multiple comparisons, statistical tests of channel-wise activation will be corrected using the false discovery rate (FDR). Functional connectivity (FC), wavelet amplitude (WA), and graph-theoretical measures calculated in NirSpark will be exported for subsequent statistical analysis using linear mixed-effects models (LMM), with Bonferroni correction applied to multiple comparisons.

Biomechanical and clinical data: Repeatedly measured sEMG, muscle tension, pressure pain threshold (PPT), VAS, and ODI outcomes will be analyzed using repeated-measures ANOVA when the data are normally distributed. For non-normally distributed repeated measurements, generalized estimating equations (GEE) will be used in Parts 1 and 3, whereas the Friedman test will be used for the low-pressure, medium-pressure, and high-pressure conditions in Part 2. Bonferroni correction will be applied where multiple comparisons are required.

Regression and correlation analyses: For outcomes showing differences across pressure levels, regression analysis will be further performed to examine how the outcomes change with increasing pressure and to identify the pressure level or range associated with a more favorable response. Linear and polynomial regression models will be compared using adjusted R^2^, Akaike information criterion (AIC), and Bayesian information criterion (BIC), and the better-fitting model will be used for interpretation. In Part 2, correlation analyses will be performed among the biomechanical measures. In Part 3, exploratory correlation analyses will be performed between changes in peripheral measures, including muscle tension and PPT, and changes in fNIRS-derived FC and WA. Pearson correlation analysis will be used to assess linear relationships between normally distributed variables, whereas Spearman rank correlation analysis will be used to assess associations between non-normally distributed variables. False discovery rate (FDR) correction will be applied to correlation analyses involving multiple comparisons.

## 3 Discussion

This study establishes a three-part research framework integrating pain stimulation, cortical hemodynamic responses, manipulation parameter optimization, and clinical efficacy evaluation. By combining functional near-infrared spectroscopy and biomechanical assessments, this study aims to investigate cortical hemodynamic responses associated with lumbar muscle strain pain, identify biomechanically favorable pressure parameters for rolling manipulation, and explore the potential association between peripheral biomechanical responses and cortical hemodynamic changes during treatment. This study may provide preliminary evidence for the standardization and further development of Tuina manipulation.

This study adopted a controlled design to investigate differences in cortical hemodynamic responses between patients with lumbar muscle strain and healthy individuals under different intensities of painful stimulation. The research approach of this experiment is somewhat consistent with current research directions on the central regulation of chronic musculoskeletal pain, and has a certain theoretical and research foundation. Previous studies have confirmed that chronic musculoskeletal pain could induce significant changes in brain structure and function, typically manifested as decreased functional connectivity in the prefrontal cortex and abnormal spontaneous brain activity (32), as well as hemodynamic abnormalities in pain-related brain regions such as the primary motor cortex and somatosensory cortex (33). These changes are consistent with the theory of central sensitization, where persistent nociceptive input leads to hyperexcitability and functional reorganization of central pain-processing pathways (34). The prefrontal cortex and its subregions exerted multiple functions in pain processing, including nociceptive localization, attention allocation, and emotional modulation, and mostly showed significant activation under painful stimulation (35–37). By using graded pain stimulation and fNIRS, this study will systematically analyze the correlation between lumbar muscle strain pain and brain activation, examining cortical response characteristics associated with lumbar muscle strain pain and identifying cortical regions associated with pain processing in lumbar muscle strain.

This study graded and screened the pressure parameters of the rolling manipulation from a biomechanical perspective, aiming to overcome the subjective limitations of traditional tuina that relies on empirical operation. Existing studies have shown that the core mechanism of massage-like manipulations is closely related to mechanotransduction: mechanical pressure act on the skin, fascia and muscle tissues, and regulate inflammatory responses and pain regulatory pathways through cellular signal transduction (38). The therapeutic effect of manipulations does not simply depend on the magnitude of force, but on the standardized combination of pressure, frequency and duration, and a stronger pressure does not necessarily lead to a better effect (38–40). Studies on tuina for peripheral induced neuropathic pain have also confirmed that appropriate manual stimulation can exert analgesic effects through peripheral anti-inflammation, regulating ion channels and altering central brain function (41). Accordingly, this study defined low, medium and high levels based on individualized pressure calibration combining VAS perception and objective pressure measurements. Optimal parameters are selected using multi-dimensional biomechanical indicators including surface electromyography and isokinetic muscle strength, and standardized monitoring of manipulation parameters is realized via a dynamic pressure distribution measurement system. It is intended to establish a quantitative correlation system of “individual pain threshold-manipulation mechanical parameters-biological effect”, so as to formulate an objective and repeatable pressure standard for the rolling manipulation and solve the key problem of the lack of quantitative specifications for clinical tuina parameters.

The application of fNIRS technology in monitoring cortical hemodynamic responses during Tuina intervention takes advantage of the unique features of this technology and provides a new methodological approach for investigating cortical responses associated with Tuina manipulation. At present, a variety of functional neuroimaging technologies have been proven to be applicable to the research of brain pain perception (42). With the advantages of portability, low cost and robustness against motion artifacts, fNIRS is more suitable for real-time and dynamic monitoring in clinical scenarios (43) and has successfully realized the quantitative detection of pain-related brain region activation in studies such as myofascial pain syndrome (44). Existing fNIRS studies related to tuina have confirmed that tuina could regulate the blood oxygen levels of brain regions such as the prefrontal cortex and sensorimotor cortex to achieve central regulation of motor nerves and pain perception (45, 46). However, fNIRS-based investigations of cortical hemodynamic responses during Tuina treatment for musculoskeletal disorders such as lumbar muscle strain remain limited. Dynamic monitoring of changes in cerebral blood oxygen before, during and after pain stimulation and rolling manipulation intervention by fNIRS could capture the real-time cortical hemodynamic response, and preliminarily characterize the cortical hemodynamic responses of rolling manipulation in the treatment of lumbar muscle strain, and evaluate the potential utility of fNIRS as an objective monitoring tool for tuina-related cortical hemodynamic responses. Continuous physiological signals such as heart rate, blood pressure, and respiration are not recorded synchronously during fNIRS acquisition; therefore, systemic and extracerebral hemodynamic contributions cannot be fully excluded. Accordingly, HbO/HbR changes in this study will be interpreted as cortical hemodynamic responses associated with the experimental conditions rather than as direct measures of neuronal activation.

The multidimensional evaluation framework, integrating biomechanical assessments, fNIRS-derived cortical hemodynamic responses, and clinical efficacy indicators, provides a comprehensive approach to explore the relationship between peripheral biomechanical responses and cortical changes associated with rolling manipulation. This framework may contribute to further understanding of the potential mechanisms underlying Tuina effects. Existing studies have confirmed that tuina manipulations could activate peripheral mechanoreceptors and transmit signals to the central nervous system through repeated therapeutic touch, which could not only induce immediate acute changes in brain function to achieve short-term analgesia and relaxation, but also drive the brain to undergo neural plastic adaptation, reshape the pain regulation circuit, and realize the lasting recalibration of central function (40, 47). Simultaneous assessment of peripheral biomechanical indicators, fNIRS-derived cortical hemodynamic responses, and clinical efficacy indicators enables exploratory analysis of the relationships among peripheral responses, cortical changes, and clinical outcomes. This approach may provide preliminary evidence for understanding the biological responses associated with rolling manipulation and contribute to the scientific interpretation of TCM Tuina.

Although this study has significant advantages in research design and technology integration, there are still potential limitations based on the current technological development and research conditions: Restricted by the inherent characteristics of fNIRS technology, it could only detect changes in cerebral cortical blood oxygen concentration and could not explore the functional changes of subcortical pain-related nuclei such as the thalamus and amygdala, which might lead to an incomplete characterization of subcortical pain-related processes (48). The study intended to screen the optimal parameters using low, medium and high force levels, but has not yet conducted an in-depth exploration of the precise force values and pressure distribution within the optimal level, and it is necessary to improve the individualized adjustment scheme of manipulation parameters in the future. The single-center study design results in a relatively high homogeneity of the enrolled population, and even if the sample size is scientifically estimated and the attrition rate is reserved, the extrapolability of the research results may still be affected to a certain extent. The case control group in this study receives health education, with the aim of evaluating the clinical effect of optimal-pressure rolling manipulation relative to health education. The present study does not separately address the effects of treatment time, physical contact, placebo effects, or other nonspecific factors. In addition, the treating practitioner cannot be blinded to the intervention being delivered, which is an inherent limitation of manual therapy trials. This potential performance bias will be mitigated by blinding the outcome assessors and data analysts.

Based on the design idea and potential limitations of this study, combined with the current research progress, follow-up extended research could be carried out in various aspects. Ultrasound elastography could be used to assess changes in lumbar soft tissue stiffness before and after rolling manipulation, providing direct biomechanical evidence for the therapeutic effects of the intervention. In addition, if it is confirmed that fNIRS could effectively detect brain function changes under rolling manipulation intervention, multi-modal neuroimaging research could be further carried out in combination with fMRI and EEG to make up for the deficiency of insufficient detection depth of fNIRS and comprehensively investigate the cortical–subcortical pain network involved in rolling manipulation responses. Machine learning technology is introduced to construct a prediction model for the curative effect of tuina in the treatment of lumbar muscle strain based on fNIRS brain function characteristics, realizing the precise evaluation of tuina treatment (32, 43). After screening out the optimal pressure parameters of rolling manipulation, multi-center, large-sample long-term follow-up clinical trials are carried out to verify the universality and long-term curative effect of the parameters, providing further evidence for evaluating the generalizability and clinical applicability of the parameters.

This hierarchical protocol integrates fNIRS and biomechanical assessments to investigate key scientific questions related to Tuina parameter optimization and cortical responses during intervention. If successful, this study will characterize cortical hemodynamic response patterns associated with LMS pain, identify biomechanically supported pressure parameters for rolling manipulation, explore the potential association between peripheral biomechanical responses and cortical hemodynamic changes during treatment, and evaluate the feasibility of applying fNIRS in Tuina research. The findings may provide preliminary evidence for the optimization of rolling manipulation parameters and a better understanding of biological responses associated with Tuina intervention.

## Data Availability

The original contributions presented in the study are included in the article/supplementary material, further inquiries can be directed to the corresponding authors.
